# Discrepancies among Scopus and Web of Science, coverage of funding information in medical journal articles: a follow-up study

**DOI:** 10.5195/jmla.2023.1513

**Published:** 2023-07-10

**Authors:** Peter Kokol

**Affiliations:** 1 peter.kokol@um.si, University of Maribor, Faculty of Electrical Engineering and Computer Science, Koroška ulica 46, 2000 Maribor, Slovenia.

**Keywords:** Research funding, funding acknowledgement, bibliometrics

## Abstract

**Objective:**

This follow-up study aims to determine if and how the coverage of funding information in Web of Science Core Collection (WoS) and Scopus changed from 2015 to 2021.

**Methods:**

The number of all funded articles published in 2021 was identified in WoS and Scopus bibliographic databases using bibliometric analysis on a sample of 52 prestigious medical journals.

**Results:**

The analysis of the number of funded articles with funding information showed statistically significant differences between Scopus and WoS due to substantial differences in the number of funded articles between some single journals.

**Conclusion:**

Due to significant differences in the number of funded articles indexed in WoS and Scopus, which might be attributed to the different protocols for handling funding data in WoS and Scopus, we would still advise using both databases to obtain and analyze funding information.

## INTRODUCTION

Research databases such as Web of Science Core Collection, Dimensions, Scopus, MEDLINE, and similar organize research information for knowledge discovery, research assessment, and research access. Bibliographic data that can be used for bibliometric analysis from these research databases is also essential to the research community. This data is currently used to assess research visibility, evolution, and regional and global collaborations through bibliometric analysis [1–3]. Most bibliometric research assessments are based on citations; other important data for this are funding information, co-authorship, publishers' and authors' location, and the number of authors. For instance, funding information has been used to predict research visibility, researchers' maturity, authors' intention to collaborate, and prolific funding agencies. Funding information has also been included in some national, institutional, or regional research policies in assessing researchers or research institutions for promotion, funding consideration, ranking, and awards [4–12]. Librarians may be asked to analyze the linkages between funding and academic publications, information sources or research topics, identify funding possibilities, to perform bibliometric analyses involving funding acknowledgment patterns identification and similar [13–15]The emergence of the COVID pandemic has increased the need for researchers to be informed about funding strategies for COVID research and there are a the number of bibliometric funding studies which analyze international cooperation in COVID research [16], funding of COVID research projects [17], and the funding of COVID vaccine development [18].

Journal indexing systems should provide accurate information because their role in the formal evaluation of scientific productivity translates into the power to steer research [19]. Web of Science and Scopus are the two most important bibliographic databases providing funding information [20,21]. Previous research has shown that there are some discrepancies between those two databases in general, for example, in the journal subject classification [22], the number of published records [23], the number of citations [24], and the document type [25]. This study is a follow up to the 2015 study on funding data differences of prestigious medical journals in WoS, Scopus, and PubMed [26] The 2015 study revealed significant differences between the number of funded articles (FAs) in those three bibliographic databases and that WoS contained the largest number of FAs. The follow-up study's objective is first to determine if and how the coverage of funding information in WoS and Scopus for the same family of medical journals changed from 2015 to 2021 and secondly to assess the differences and overlap of funding information of those two databases in 2021.

## METHODOLOGY

In the original study, the authors analyzed funding information for articles published in three prestigious families of medical journals: The Lancet, Journal of American Medical Association (JAMA), and British Medical Journal (BMJ) indexed in WoS and Scopus bibliographical databases. The selection of the above three families were chosen because they are highly regarded, well known, and impactful in terms of citations received. PubMed funding data can only be exported in NBIB format (the NBIB file is a bibliographic citation file saved in the PubMed format), which could not be analysed in the form as it can be with the other two databases. Additionally, since 2016 WoS has included Medline funding data in its bibliographic records, and all the analyzed journals are indexed in both PubMed and WoS; we didn't include PubMed in the present study. The information if an article was funded was obtained from the Funding organization field in WoS and the Funding sponsor field in Scopus. Two types of corpora, one for FAs and one for all articles for each database, were created for articles published in 2021. Search strings used are shown in Table 1.

**Table 1:** Search strings used in the study. A list of all possible funding organizations and sponsors was formed using the wildcard character (*).

Bibliographic database	Search string for All articles corpora	Search string for FAs corpora
Web of Science (WoS)	SO = (JAMA* or BMJ* or Lancet*) and PY = 2021	SO = (JAMA* or BMJ* or Lancet*) and PY = 2021 and FO = (a* or b* or c* or d* or e* or f* or g* or h* or i* or j* or k* or l* or m* or n* or o* or p* or q* or r* or s* or t* or u* or v* or z* or x* or y* or w* or 1* or 2* or 3* or 4* or 5* or 6* or 7* or 8* or 9* or 0*)
Scopus	SRCTITLE(Lancet or BMJ or JAMA) and PUBYEAR = 2021	SRCTITLE(Lancet or BMJ or JAMA) and PUBYEAR = 2021 and FUND-SPONSOR(a* or b* or c* or d* or e* or f* or g* or h* or i* or j* or k* or l* or m* or n* or o* or p* or q* or r* or s* or t* or u* or v* or z* or x* or y* or w* or 1* or 2* or 3* or 4* or 5* or 6* or 7* or 8* or 9* or 0*)

The metadata regarding the number of all articles and the number of FAs were downloaded from WoS and Scopus using the export functions available through the database. Since the distributions of the number of all articles and the number of FAs in both databases were not parametric, as shown by the Kolmogorov Smirnov test, we used the Wilcoxon Signed Rank test to compare them. Statistical analysis was performed in SPSS Version 27.

## RESULTS

According to the search, the same 52 journals were indexed in both Scopus and WoS in 2021. Of the 26,048 articles identified in Scopus, 9,079 (34.85%) were funded, compared with 10,162 (36.20%) from the 28071 articles identified in WoS. The average difference between single journals was 11.72%; the largest difference in the number of identified articles were observed in BMJ, where 1,760 articles were identified in Scopus and 3,114 articles in WoS.

The Wilcoxon Signed Ranks test showed that the difference in the number of all papers identified is significant (Z=-2.911, p=0.004). Contrarily, the number of FAs detected in both databases did not differ significantly between Scopus and Web of Science (Z=-1.491, p=0.136). The largest differences in the percentages of FAs in favor of WoS were observed in the journals BMJ Supportive and Palliative Care (34.04%), JAMA Network Open (33.29%), and BMJ Open Sport and Exercise Medicine (22.12%). The journals where we identified the largest differences between Scopus and WoS in favor of Scopus were JAMA Journal of the American Medical Association (29.88%), Lancet Rheumatology (18.08), and JAMA Neurology (13.13%). The journals where FAs were the most similar were Lancet Neurology (0.59%), BMJ Case Reports (1.26%), and Lancet Public Health (2.28%). There were 26 journals where more FAs were identified in Scopus and 26 journals where more FAs were identified in WoS.

In both Scopus and WoS, the largest percentage of FAs were identified in BMJ Open Diabetes Research and Care (Scopus: 64.63%, WoS: 85.51%), BMJ Open (Scopus: 60.82%, WoS: 77.38%), and BMJ Global Health (Scopus: 58.67%, WoS: 70.45%).

**Table 2:** Number of all and funded articles published in 2021 in three prestigious families of medical journals indexed in WoS and Scopus

	Source title	Scopus			WoS		
		All articles	Funded articles	% of FAs	All articles	Funded articles	% of FAs
1	BMJ	1760	259	14.72	3114	271	8.70
2	BMJ Case Reports	3460	57	1.65	3201	93	2.91
3	BMJ Evidence Based Medicine	142	33	23.24	170	61	35.88
4	BMJ Global Health	617	362	58.67	555	391	70.45
5	BMJ Health And Care Informatics	74	27	36.49	67	34	50.75
6	BMJ Leader	94	16	17.02	126	40	31.75
7	BMJ Military Health	193	9	4.66	256	62	24.22
8	BMJ Neurology Open	37	10	27.03	39	19	48.72
9	BMJ Open	4410	2682	60.82	3965	3068	77.38
10	BMJ Open Diabetes Research And Care	229	148	64.63	214	183	85.51
11	BMJ Open Gastroenterology	95	32	33.68	91	42	46.15
12	BMJ Open Ophthalmology	103	44	42.72	102	57	55.88
13	BMJ Open Quality	242	66	27,27	229	113	49.34
14	BMJ Open Respiratory Research	165	77	46.67	162	105	64.81
15	BMJ Open Sport and Exercise Medicine	129	40	31.01	128	68	53.13
16	BMJ Paediatrics Open	147	44	29.93	149	61	40.94
17	BMJ Quality and Safety	156	55	37.16	213	125	58.69
18	BMJ Sexual and Reproductive Health	87	21	24.14	133	61	45.86
19	BMJ Simulation and Technology Enhanced Learning	135	25	18.52	135	39	28.89
20	BMJ Supportive and Palliative Care	223	24	10.76	337	151	44.81
21	JAMA Cardiology	369	189	51.22	400	146	36.50
22	JAMA Dermatology	310	101	32.58	324	98	30.25
23	JAMA Internal Medicine	599	266	44.41	631	173	27.42
24	JAMA Journal of The American Medical Association	1288	567	44.02	1584	224	14.14
25	JAMA Network Open	2232	638	28.58	2108	1304	61.86
26	JAMA Neurology	288	153	53.13	300	120	40.00
27	JAMA Oncology	478	223	46.65	522	184	35.25
28	JAMA Ophthalmology	382	142	37.17	408	133	32.60
29	JAMA Otolaryngology Head and Neck Surgery	261	76	29.12	301	72	23.92
30	JAMA Pediatrics	483	189	39.13	541	177	32.72
31	JAMA Psychiatry	250	142	56.80	263	142	53.99
32	JAMA Surgery	462	128	27.71	523	120	22.94
33	Lancet	1233	405	32.85	1595	434	27.21
34	Lancet Child and Adolescent Health	207	55	26.57	208	67	32.21
35	Lancet Diabetes and Endocrinology	176	80	45.45	174	62	35.63
36	Lancet Digital Health	138	59	42.75	132	68	51.52
37	Lancet Gastroenterology and Hepatology	247	86	34.82	320	72	22.50
38	Lancet Global Health	417	140	33.57	425	167	39.29
39	Lancet Haematology	204	79	38.73	218	76	34.86
40	Lancet Healthy Longevity	142	64	45.07	199	68	34.17
41	Lancet HIV	154	73	47.40	145	82	56.55
42	Lancet Infectious Diseases	595	199	33.45	625	167	26.72
43	Lancet Microbe	124	58	46.77	175	68	38.86
44	Lancet Neurology	282	96	34.04	272	91	33.46
45	Lancet Oncology	522	201	38.51	525	162	30.86
46	Lancet Planetary Health	178	68	38.20	174	71	40.80
47	Lancet Psychiatry	311	89	28.62	329	87	26.44
48	Lancet Public Health	172	79	45.93	168	81	48.21
49	Lancet Regional Health Europe	236	101	42.80	245	91	37.14
50	Lancet Regional Health Western Pacific	220	124	56.36	234	110	47.01
51	Lancet Respiratory Medicine	380	151	39.74	369	136	36.86
52	Lancet Rheumatology	210	93	44.29	248	65	26.21

When we compare the 2015 study data to its 2021 follow up study data, we see that the number of journals covered in both studies almost doubled, from 28 in 2015 to 52 in 2021. The number of FAs increased significantly in both databases, from 7.7% in the 2015 study to 34.9% in Scopus and from 29.2% to 36.2% in WoS. While in 2015, there was a significant statistical difference in the number of FAs between Scopus and WoS, and no difference in the number of all articles, the situation was the opposite in 2021; there was no significant statistical difference in the number of FAs between Scopus and WoS, and a considerable difference in the number of all articles. Hoverer in both studies there were more FAs found in WoS then in Scopus.

The most frequent types of FAs identified in WoS were in articles (71%), reviews (11%), editorials (8%), and letters (7%), whereas the most frequent types of FAs in Scopus were in articles (73%), reviews (13%), letters (11%), and editorials (1%). The main difference between both databases is the percentages of editorials and letters, the first having more funding acknowledgments in WoS and the second in Scopus. In the 2015 study, all FAs in Scopus were articles, while in 2021, articles represented 59% of FAs.

The analysis of the most prolific funding organizations in both databases is shown in Table 3. We can see that both lists differ significantly. Among funding organizations, the United States Department of Health and Human Services, European Commission, and UK Research Innovation are mentioned considerably more frequently in WoS. In contrast, National Institute for Health Research, Welcome Trust, and pharmaceutical organizations are more frequent in Scopus. To be able to perform this comparison we had to align slight differences in funding organization naming between both two databases. The process was done manually.

**Table 3:** Funding organizations that appear at least 300 times in either WoS or Scopus

Funding Agencies	WoS	Scopus
United States Department of Health and Human Services	2113	994
National Institutes of Health NIH USA	1971	2050
European Commission	914	392
UK Research Innovation	743	489
Medical Research Council UK	599	537
National Institute for Health Research	575	1056
Welcome Trust	447	607
National Health and Medical Research Council of Australia	369	454
National Natural Science Foundation of China	364	342
Canadian Institutes of Health Research	333	336
Bill Melinda Gates Foundation	292	410
National Cancer Institute	259	338
National Institute on Aging	242	340
Astra Zeneca	107	554
Pfizer	83	654
Merck	74	462
Roche	55	454
Novartis	61	453
Bristol-Myers Squibb	55	368
Glaxo Smith Cline	52	356
Boehringer Ingelheim	48	308

## DISCUSSION

The difference in the percentage of FAs between Scopus and WoS in the family of prestigious medical journals has reduced since 2015 from 21.5% to 1.4%. in 2021. However, the difference is still statistically significant due to sometimes large differences between single journals. Furthermore, there are only 25 journals where the difference between percentages of FAs in both databases is less than 10%, mainly in the cases where percentages of FAs are larger in Scopus.

According to our analysis, one of the differences between WoS and Scopus FAs is the larger share of acknowledgments appearing in documents labeled as editorials in WoS. But we also believe that the main reason is the handing/acquiring of funding data by both databases. According to the Web of Science Group [27], WoS started to supplement funding information with grant agencies from Researchfish and Medline in 2016 and simultaneously started to unify the funding data. On the other hand, Scopus comprehensively cover grants from the United States, United Kingdom, pan European bodies, and some other selected funding organizations around the globe, based on the Founder Registry, which was facilitated by CrossRef, and Elsevier is one of the founders [28].

While recently, bibliographic databases have become leading providers of publication metadata for research assessment [9,20] and research grants performance and monitoring [29,30], our study implies that selecting the only one of these databases might produce misleading results. For example, a funding agency might evaluate their impact by analyzing the number of FAs in publications and over or underestimate the impact by selecting the wrong database. Similarly, a research institution's human relations manager might use this data to find or consider suitable candidates and may not have an accurate representation of how they have used funding to support their research. On a more individual level, a researcher seeking funding for their research project might submit a proposal to an agency that does not financing their research topic. We would, therefore, advise researchers, librarians, grant administrators seeking funding information related to medical topics, funding bodies, or research organizations to use both databases to obtain more reliable information about funding data and patterns.

## STUDY LIMITATIONS

The study has potential limitations. The search string might not have captured all FAs, the same string was used in the original study and may not capture funding organization names that start with something other than a letter. The analysis was performed on a sample of 52 medical journals, among more than 1000 indexed in both WoS and Scopus in 2021, so the generalization of the results might be limited.

## Data Availability

For legal reasons, data from Clarivate Web of Science and Scopus cannot be made openly available.
